# Diversification, loss, and virulence gains of the major effector AvrStb6 during continental spread of the wheat pathogen *Zymoseptoria tritici*

**DOI:** 10.1371/journal.ppat.1012983

**Published:** 2025-03-31

**Authors:** Ana Margarida Sampaio, Sabina Moser Tralamazza, Faharidine Mohamadi, Yannick De Oliveira, Jérôme Enjalbert, Cyrille Saintenac, Daniel Croll

**Affiliations:** 1 Laboratory of Evolutionary Genetics, Institute of Biology, University of Neuchâtel, Neuchâtel, Switzerland; 2 Arvalis - Institut du Végétal, Station expérimentale, Boigneville, France; 3 Université Paris-Saclay, INRAE, CNRS, AgroParisTech, GQE-Le Moulon, Gif-sur-Yvette, France; 4 Université Clermont Auvergne, INRAE, GDEC, Clermont-Ferrand, France; University of Cologne, GERMANY

## Abstract

Interactions between plant pathogens and their hosts are highly dynamic and mainly driven by pathogen effectors and plant receptors. Host-pathogen co-evolution can cause rapid diversification or loss of pathogen genes encoding host-exposed proteins. The molecular mechanisms that underpin such sequence dynamics remains poorly investigated at the scale of entire pathogen species. Here, we focus on *AvrStb6*, a major effector of the global wheat pathogen *Zymoseptoria tritici*, evolving in response to the cognate receptor *Stb6*, a resistance widely deployed in wheat. We comprehensively captured effector gene evolution by analyzing a global thousand-genome panel using reference-free sequence analyses. We found that AvrStb6 has diversified into 59 protein isoforms with a strong association to the pathogen spreading to new continents. Across Europe, we found the strongest differentiation of the effector consistent with high rates of *Stb6* deployment. The *AvrStb6* locus showed also a remarkable diversification in transposable element content with specific expansion patterns across the globe. We detected *AvrStb6* gene losses and evidence for transposable element-mediated disruptions. We used virulence datasets of genome-wide association mapping studies to predict virulence changes across the global panel. Genomic predictions suggested marked increases in virulence on *Stb6* cultivars concomitant with the spread of the pathogen to Europe and the subsequent spread to further continents. Finally, we genotyped French bread wheat cultivars for *Stb6* and monitored resistant cultivar deployment concomitant with *AvrStb6* evolution. Taken together, our data provides a comprehensive view of how a rapidly diversifying effector locus can undergo large-scale sequence changes concomitant with gains in virulence on resistant cultivars. The analyses highlight also the need for large-scale pathogen sequencing panels to assess the durability of resistance genes and improve the sustainability of deployment strategies.

## Author summary

Interactions between plants and their specialized pathogens are often mediated by a sophisticated molecular dialogue. Effectors produced by pathogens serve to manipulate the host but may also be used by the host to trigger defense mechanisms upon recognition. Deploying plants carrying a resistance gene against a specific effector could lead to rapid adaptation in the pathogen. Here, we unraveled such dynamics at the scale of the global distribution range of the fungal wheat pathogen *Zymoseptoria tritici*. The effector is encoded by the gene *AvrStb6* located in a polymorphic region of a chromosome near the telomere. We find selfish elements (*i.e.,* transposable elements) repeatedly inserted nearby the gene, which has likely facilitated the rapid sequence evolution. The effector diversified among continents, and we could predict that the sequence changes likely helped escape recognition by the host receptor. Our study provides one of the most comprehensive views how a crop pathogen diversified a major effector in response to host resistance factors. Such studies facilitate devising more durable deployment strategies of host resistance in order to maintain crop yield.

## Introduction

Interactions between plant pathogens and their host is a highly dynamic process, mediated by various components, including pathogen effectors (*i.e.,* avirulence factors, *Avr*) and resistance (*R*) plant genes. Effector genes encode secreted molecules capable of modulating host plant metabolism or suppress plant immune responses, thereby are crucial for successful host infection [1]. In turn, *R* genes encode proteins that can recognize specific pathogen effectors, particularly those encoded by *Avr* genes, subsequently triggering an immune response [2,3]. In this gene-for-gene model, the presence of *R* genes imposes selection pressure on pathogens carrying recognized effectors [2]. This favors effector mutations preventing recognition by purging avirulent protein variants [4,5]. Mechanisms include transposon insertions [6], repeat-induced mutations (RIP) [7] or even complete loss of recognized effector. Being beneficial, those mutations often spread rapidly through the pathogen population [8].

The dynamics of effector evolution are often influenced by their chromosomal sequence environment. They can be located in either accessory chromosomes enriched in repetitive sequences [9] or transposable element (TE) rich core chromosome compartments [9,10]. Repeat proximity facilitates effector sequence diversification and, hence, increases mutations available for adaptation to an evolving host [11,12]. Such rapid virulence evolution was described in the rice pathogen *Magnaporthe oryzae* due to effector localization in highly repetitive subtelomeric region. Mutations in *AVR-Pita* effectors, such as point mutations, insertions and deletions, have enabled the fungus to evolve avoiding triggering immune response [13]. Furthermore, this effector has undergone multiple translocations associated with virulence evolution [14]. The transposition of TEs can disrupt effector coding sequences or alter their regulation. In *M. oryzae*, the insertion of a Mg-SINE TE in the *AvrPi9* gene led to loss-of-function of the effector [15], while in *Verticillium dahlia*, a TE insertion inactivated the *Ave1* effector [16], both probably enabling the pathogen to escape host recognition. In addition, repeat-induced point (RIP) mutations, a defense mechanism against TEs, can impact effector diversification and enhance virulence. In the fungal pathogen *L. maculans*, the emergence of virulence alleles in *AvrLm1* was driven by RIP mutations, resulting in a non-functional locus and host resistance breakdown [17]. Furthermore, leakage of RIP into neighboring regions contributed to the diversification of effector genes while retaining functionality [7]. Taken together, effector gene diversification and TE dynamics of the surrounding regions are key factors to assess the evolutionary potential of the pathogen. However, how the effectors evolved in response to strong host selection pressure across and within species remains poorly understood.

*Zymoseptoria tritici*, the causal agent of septoria tritici blotch (STB), is one of the major fungal foliar diseases of wheat-growing areas worldwide [18]. The spread of the pathogen has been tightly associated with the origins of wheat cultivation [19]. With its center of origin located in the Middle East, *Z. tritici* initially colonized North Africa and Europe, and later migrated to the Americas and Oceania [20]. Population genomic analyses based on single nucleotide polymorphism (SNP) analyses of a global panel of >1000 sequenced genomes identified eleven well-supported genetic clusters with most being restricted to individual continents. In the Middle East, two distinct clusters distinguished isolates from Iran and Israel. Isolates collected in Northern Africa and Europe were also represented by two different clusters, while Australian and New Zealand isolates were grouped into three clusters. North American *Z. tritici* populations grouped in two clusters along a North-South separation, while in South America two clusters split pathogen diversity along the Andes [20]. Even though continental divisions reflect historic restrictions in gene flow, a significant fraction of isolates mismatched the prevalent genetic cluster on the continent consistent with recent migration events. Europe was the strongest source for such events across continents [20]. Beyond changes in genetic diversity, *Z. tritici* has also undergone shifts in transposable element (TE) content, with the most recently colonized areas (Americas and Oceania) showing higher numbers of genome-wide TE insertions and incipient expansions in genome size likely associated by the loss of RIP activity [20,21]. TEs cover 16.5-24% of the genome and are often located near genes involved in host-interactions [22]. TEs activity can also be directly associated with effector gene expression and virulence [23–25].

The first avirulence effector to be cloned in *Z. tritici* was *AvrStb6* [26,27], which plays a dominant role in immunity evasion due to the prevalence of the corresponding resistance gene. The effector is encoded in a highly polymorphic subtelomeric region of chromosome 5 surrounded by TEs [8,27,28]. *AvrStb6* is recognized by the plant wall-associated receptor-like kinase *Stb6* [29], present in many wheat cultivars worldwide, as it has been frequently used in breeding programs to control septoria tritici blotch (STB), though its usage varies across different regions [30,31]. Given the broad deployment of *Stb6*, *Z. tritici* is expected to experience significant host selection pressure to escape recognition. Indeed, *AvrStb6* shows high haplotype diversity across the world [32–34] with a likely absence of the originally described avirulent isoform among recently collected isolates, consistent with efficient counter-selection against avirulent haplotypes. Rare premature stop codons have been identified in the coding sequence [32,33], however no complete loss of *AvrStb6* has been documented yet, which was interpreted as evidence for essential but not yet known role of *AvrStb6* [32]. Given the high plasticity of the subtelomeric region surrounding *AvrStb6* [8], active TEs could continue to reshape sequence diversity in extant populations.

To address how *AvrStb6* diversification occurred during continental spread, we aimed to provide a large-scale population-genomics informed view how *AvrStb6* and the surrounding regions evolved in response to varying selection pressure imposed by past release of *Stb6* in wheat varieties. For that, we recapitulated *AvrStb6* evolution in a thousand-genome panel of *Z. tritici* covering key regions associated with the historical dissemination of wheat cultivation. We combined reference-genome data with short-read sequencing to validate key insights about the evolution of the locus and tracked insertion dynamics of TEs using a newly established high-quality TE library. To connect *AvrStb6* evolution to the deployment of cognate wheat cultivars, we first used genomic prediction to assess virulence trait evolution across the global dataset and, second, tracked the predicted virulence gains across a European country in conjunction with monitoring wheat cultivar deployment.

## Results

### *Global AvrStb6* genetic diversity

**Fig 1:**
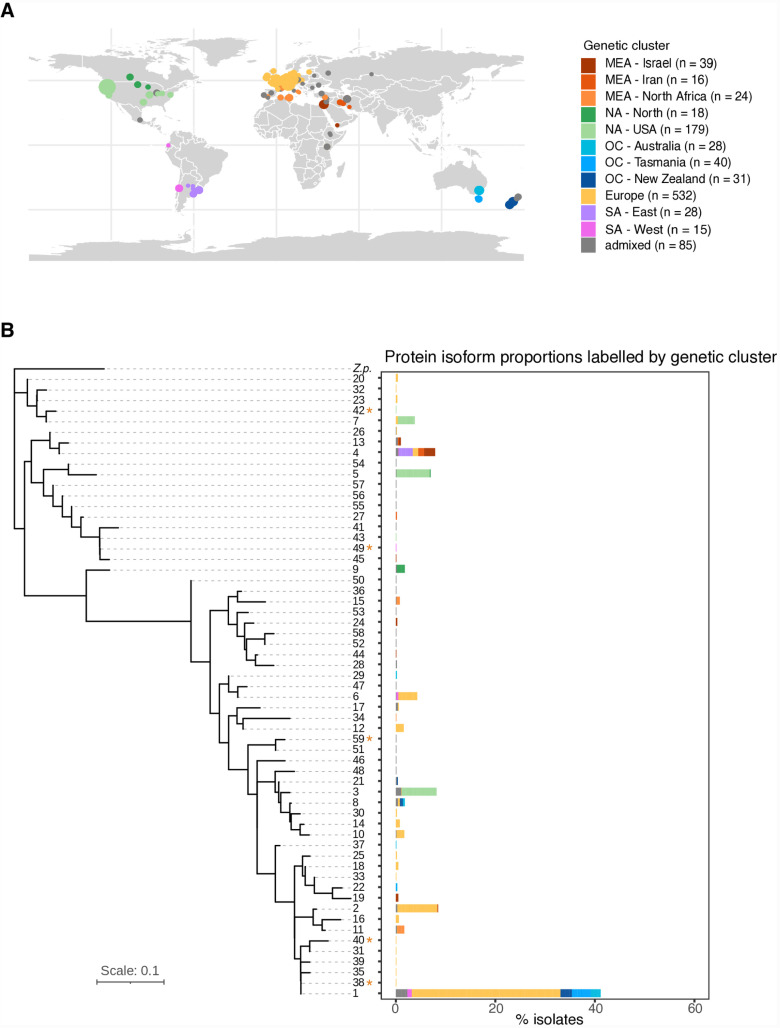
Global panel of *Zymoseptoria tritici* isolates sampled across continents and AvrStb6 diversification. A) Geographical distribution of the 1035 analyzed isolates colored by the 11 assigned genetic clusters (20). Circle sizes are proportional to the population size sampled per location. World map created with the R package *rnaturalearth* (base: Natural Earth v4.1.0 - https://www.naturalearthdata.com/downloads/110m-physical-vectors). B) Maximum likelihood phylogenetic tree of AvrStb6 protein isoforms rooted by the *Z. pseudotritici* (*Zp*) closest sister species isoform. Truncated isoforms with premature stop codon are marked with an asterisk. The barplot shows the frequency of isoforms and their composition based on genetic clusters assigned to the isolates.

The spread of the fungal wheat pathogen *Z. tritici* from its origin in the Fertile Crescent to other continents has been assessed based on a global panel of >1000 sequenced genomes [20]. Genome-wide polymorphism analyses identified eleven genetic clusters tracking the historic spread of wheat cultivation across the world [20]. *Z. tritici* emerged in the Middle East and initially colonized North Africa and Europe. More recent migration events introduced the pathogen to the Americas and Oceania. Wheat cultivars carrying the *Stb6* resistance genes are globally distributed [31]. To unravel the evolutionary trajectory of *AvrStb6* during global expansion, we analyzed the same thousand-genome panel for *AvrStb6* gene variants (Fig 1A). We searched draft genome assemblies generated for 1035 isolates sampled across the world for alleles of *AvrStb6*. We identified 1001 assemblies containing single matching alleles, excluding sequences of potentially truncated *AvrStb6* copies. The draft assemblies presented reasonable contiguity with the N50 (length of the shortest contig for which longer length contigs cover at least 50% of the assembly) ranging between 2215 and 215,440 bp (S2 Table). Intact *AvrStb6* copies were recovered ranging from 361-365 bp in length representing a total of 103 nucleotide sequence haplotypes. The encoded protein sequences ranged from 81-82 amino acids for 59 distinct isoforms (S3 Table). The most frequent isoform, labelled here as isoform 1 matches isoform I02 identified in previous work [33] (S3 Table). Isoform 1 was shared by 41% of the collected isolates. On the contrary, 49 isoforms were each represented by less than 1% of the analyzed isolates. Isoform 6, identified among others in the reference isolate IPO323, was shared by 4.2% of all isolates. We found no correlation between isoform frequencies and genetic cluster identity (Fig 1B). Three isolates from the European cluster, and two isolates from the American clusters (North America – USA and South America – West) carried a premature stop codon (S4 Table). The stop codon position was variable and ranged from the 5^th^ to the 46^th^ amino acid position. Nevertheless, amino acid sequences after the premature stop codon remained mostly conserved compared to the reference genome IPO323 *AvrStb6* haplotype (isoform 6).

We reconstructed the phylogenetic relationships among isoforms to recapitulate the effector diversification. Isolates from Middle East (Israel and Iran) carried isoforms similar to the AvrStb6 isoform recovered from the closest sister species *Z. pseudotritici*. On the contrary, isoform 1, the most frequent isoform in European and Oceanian clusters, was one of the most divergent isoforms to *Z. pseudotritici*, showing that a major transition in *AvrStb6* haplotypes occurred following European colonization (Figs 1B, S1). Additional European and Oceanian isoforms, along with Northern African isoforms, clustered closely together with the most divergent isoform*.* Isolates from the North America – USA cluster presented the most distinct set of isoforms, with two isoforms clustering together with Middle East isoforms, while another isoform revealed to be more divergent. South America – East isolates presented the same isoform as most of the Middle East isolates (isoform 4), while South America - West isolates revealed to be more divergent (Fig 1B). The five protein isoforms with premature stop codons were found across all major branches of AvrStb6 diversification (Fig 1B). Altogether, these findings indicate that *AvrStb6* diversification occurred most prominently in Europe and by this likely impacting effector trajectories in subsequently colonized continents, namely Oceania.

### Transposable element dynamics at the *AvrStb6* locus

**Fig 2:**
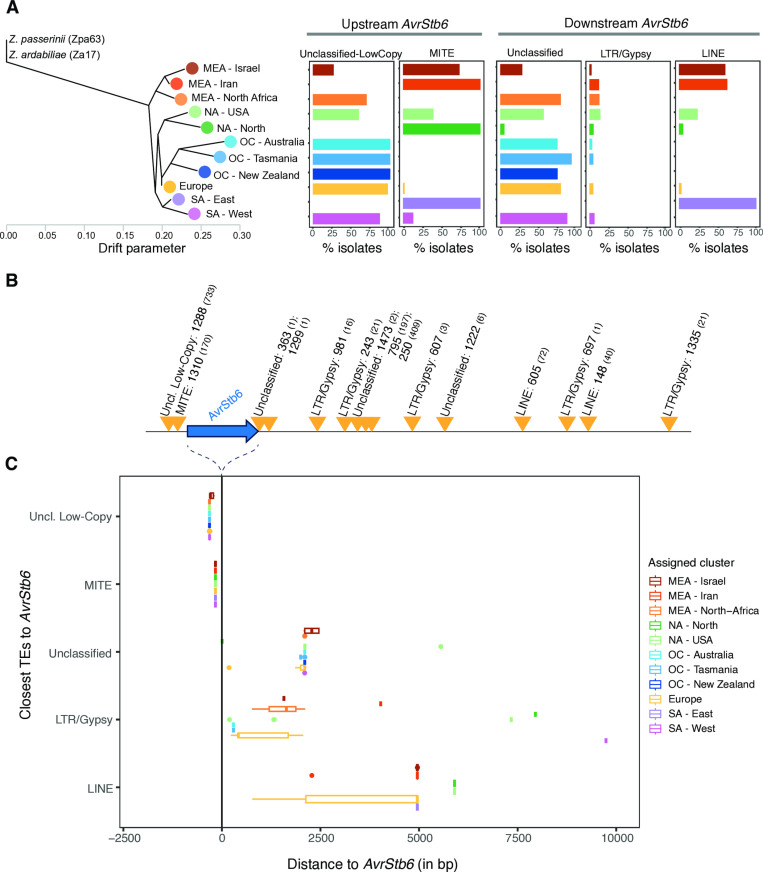
Insertion frequencies of transposable elements (TEs) 10 kb up and downstream of *AvrStb6* in the thousand-genome panel. A) TE insertion frequencies for the most frequent TE superfamilies according to the genetic clusters assigned to the isolates. The population tree was generated based on Feurtey et al. (20). B) Schematic representation illustrating the average distance of TE copies grouped by superfamilies. The number after TE superfamily identifiers represents the family number and the number within parentheses represents the number of *Z. tritici* isolates having the respective TE. C) Variation in distance between specific TEs and *AvrStb6* among isolates*.* Distance variation is summarized by TE superfamily (or unclassified TEs) for each genetic cluster. The line at zero basepairs represents the *AvrStb6* position. Unclassified low-copy TE refers to ZymTri_2023_family_1288; MITE refers to ZymTri_2023_family_1310; LTR/Gypsy refers to ZymTri_2023_family_1335, 243, 607, and 981; Unclassified TE refers to ZymTri_2023_family_1299, 1473, 250, 363, 697, 795; and LINE refers to ZymTri_2023_family_1222, 148, and 605.

TEs tend to be located closer to effector genes compared to other genes in the genome across diverse fungal pathogens. Hence, TEs have a significant potential to act as regulators of effector genes. *AvrStb6* is located in a gene-poor and TE-rich region close to a telomeric end of chromosome 5 (69,019–69,383 bp in the IPO323 reference genome). To investigate patterns of TE dynamics near *AvrStb6,* we analyzed contigs encoding *AvrStb6* among different isolates to screen for the presence of TE sequences in an interval covering 10 kb up- and downstream of the gene. Upstream of *AvrStb6*, the two most frequently inserted TE superfamilies included a miniature inverted-repeat transposable element (MITE; ZymTri_2023_family_1310) and an unclassified low-copy TE (ZymTri_2023_family_1288), with the latter one found in 796 (79.5%) of isolates. In contrast, populations sampled near the centre of origin (Middle East, Israel and Iran) as well as the South American cluster (SA-East) carried most frequently a MITE (Fig 2A). Isolates from both the European and Oceanian clusters (Australia, Tasmania and New Zealand) carried the unclassified TE most frequently (Fig 2A). Remarkably, all Oceanian clusters carried exclusively the unclassified TE near *AvrStb6*. The MITE ZymTri_2023_family_1310 was at 163 bp from the start of the coding sequence in all the 170 isolates among different population clusters where this TE has been found (Fig 2B and C) consistent with a single, recent insertion event. This TE is also the most detected TE close to *AvrStb6* (Fig 2B and C; S5 Table). Downstream of *AvrStb6*, we identified distinct insertions by TEs from different superfamilies: LINE retrotransposons (ZymTri_2023_family_1222, 148, and 605), retrotransposons LTR/Gypsy (ZymTri_2023_family_1335, 243, 607, and 981), as well as unclassified TEs (ZymTri_2023_family_1299, 1473, 250, 363, 697, 795). Downstream of *AvrStb6*, inserted TEs were at variable distance to *AvrStb6* (Fig 2B). A single isolate (STnnJGI_SRR7073594, from North America - North) carried an unclassified TE (ZymTri_2023_family_363) just at 8 bp downstream of *AvrStb6*, while 21 isolates from the North America - USA cluster carried as the closest downstream TE a retrotransposon LTR/Gypsy 7339 bp away from *AvrStb6* (Fig 2B; S5 Table). We assessed whether TE insertions were associated with *AvrStb6* expression variation under axenic culture conditions for a subset of the thousand-genome panel [35]. However, *AvrStb6* showed no meaningful expression variation outside of the plant host, hence associations with TE insertions remain inconclusive.

Overall, TEs downstream were found at larger and more variable distances to *AvrStb6* compared to upstream TEs (Fig 2B and C; S5 Table). Furthermore, both close up- and downstream TEs were partially degraded, lacking full-length sequences, suggesting that TEs were inactivated likely in recent history. Populations from the Middle East (Israel and Iran) both carry LINE retrotransposons as the most frequent TE (Fig 2A). Regions outside of the centre of origin largely carried unclassified TEs except for the South America (East) cluster, which is sharing TE patterns with isolates from the centre of origin. In conjunction, the TE insertion analyses show that TE associations with *AvrStb6* underwent significant shifts as the pathogen spread from the Middle East to North-Africa, Europe and later introductions to the Americas and Oceania.

### Rare deletion mutants at the *AvrStb6* locus across the species range

**Fig 3:**
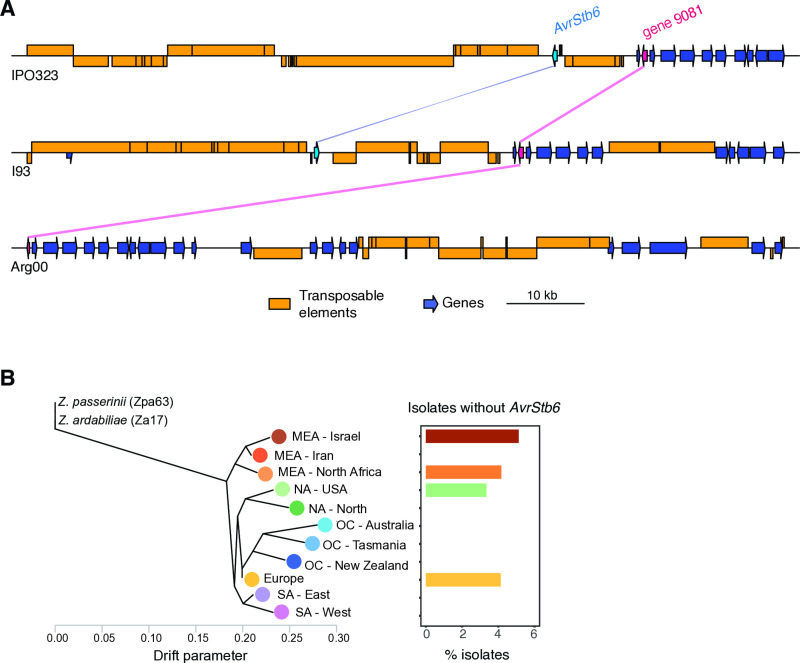
Evidence for *AvrStb6* loss. A) Synteny plot between the telomeric ends and the *AvrStb6* locus on chromosome 5 of the IPO323 reference genome, I93 (USA) and Arg00 (Argentina). Colored lines between chromosomes indicate homologous regions. B) Percentage of isolates missing *AvrStb6* per genetic cluster.

We analyzed whether loss-of-function mutants for *AvrStb6* could include gene deletions in addition to premature stop codons. We first inspected the *AvrStb6* region in the species pangenome represented by a set of 19 reference-quality genomes*,* covering all major wheat producing areas [22]. We detected *AvrStb6* alleles in all pangenome isolates except for the Argentinian isolate Arg00. In contrast to the canonical reference IPO323, the Arg00 chromosome 5 appeared truncated near the neighbouring gene downstream of *AvrStb6* (gene_9081 [36]; Fig 3), indicative of a complete loss of *AvrStb6*. To assess potential assembly artefacts in the Arg00 genome, we used raw PacBio long reads generated for Arg00 to align against the reference IPO323. The read coverage on chromosome 5 was supporting the fact that both *AvrStb6* locus as well as the neighboring subtelomeric region were missing (S2 Fig). Among the 18 pangenome isolates carrying *AvrStb6*, the isolate I93 from Indiana (USA) carried an inverted *AvrStb6* coding sequence without affecting neighboring genes (Fig 3A). As *AvrStb6* was found missing in a reference-quality genome, we screened for potential additional losses in the thousand-genome panel searching draft genome assemblies. As expected, *AvrStb6* is present in a large majority of isolates, however a small number of isolates showed patterns consistent with deletions such as gene truncation or complete absence of the *AvrStb6* coding sequence. Overall, 34 isolates (3.3%) carried no or no intact *AvrStb6*, of which 23 isolates completely lacked homology and 11 with evidence for a partial deletion (S6 Table).

We further investigated evidence for *AvrStb6* loss in the same panel of isolates using copy-number variation (CNV) calls [37]. Precision of CNV calling was found to be optimal in 1-kb windows, hence we investigated *AvrStb6* based on the 69–70 kb interval on chromosome 5. Most of the isolates identified as lacking or carrying a truncated *AvrStb6* gene based on draft assemblies (30 out of 34) showed reduced or no sequence coverage in the CNV analyses, as expected (S6 Table). Isolates without *AvrStb6* were distributed across most genetic clusters (Fig 3B). Next, we analyzed genomes with partial *AvrStb6* sequences to identify potential TE sequences at the synteny breakpoints. We detected two isolates out of 11 with a partial sequence *AvrStb6* sequence and a truncated TE sequence inserted into the locus resulting in a sequence rearrangement. In the European isolate ST16CH_1P7, a DNA/PIF-Harbinger TE fragment was overlapping with the 5’ region of the *AvrStb6* coding sequence replacing a 170 bp segment of the gene. In a second European isolate, 07STF058, a LINE retrotransposon was overlapping with the 3’ end of the truncated *AvrStb6* sequence. Interestingly, this LINE retrotransposon belongs to a family (ZymTri_2023_family_605) detected as one of the physically closest TE families downstream of *AvrStb6* (Fig 2B). Taken together, *AvrStb6* loss and truncation occurs at low frequency in *Z. tritici* populations and at least some of the loss-of-function variants were likely caused by TE-mediated sequence rearrangements.

### Genomic prediction of virulence underpinned by *AvrStb6* variation

Our next objective was to investigate whether *AvrStb6* haplotypes evolved to become more virulent within the species. Gathering phenotypic data from infections is challenging at scale. Hence, we performed genomic predictions parametrized by genome-wide association mapping studies. We used a mapping population consisting of 103 isolates, which was originally designed to identify *AvrStb6* [27]. A subset of 87 isolates were overlapping with the thousand-genome panel of the present study. Despite the relatively small GWAS panel size, the genomes encode 10 out of the 59 previously identified *AvrStb6* isoforms, including four of the seven most frequent isoforms and covering the breadth of the phylogenetic tree (Fig 1B). Zhong et al. [27] performed virulence assays on three *Stb6* cultivars Cadenza, Shafir, and Caphorn. Phenotypic readouts included green leaf area percentage, necrotic leaf area percentage, and percentage of leaf area containing pycnidia in the inoculated area [27]. The phenotypic dataset covered a broad spectrum of virulence, ranging from isolates with no lesion induction to complete coverage of leaves by lesions. Taken together, the GWAS panel includes both genetically diverse and globally representative *AvrStb6* haplotypes.

**Fig 4:**
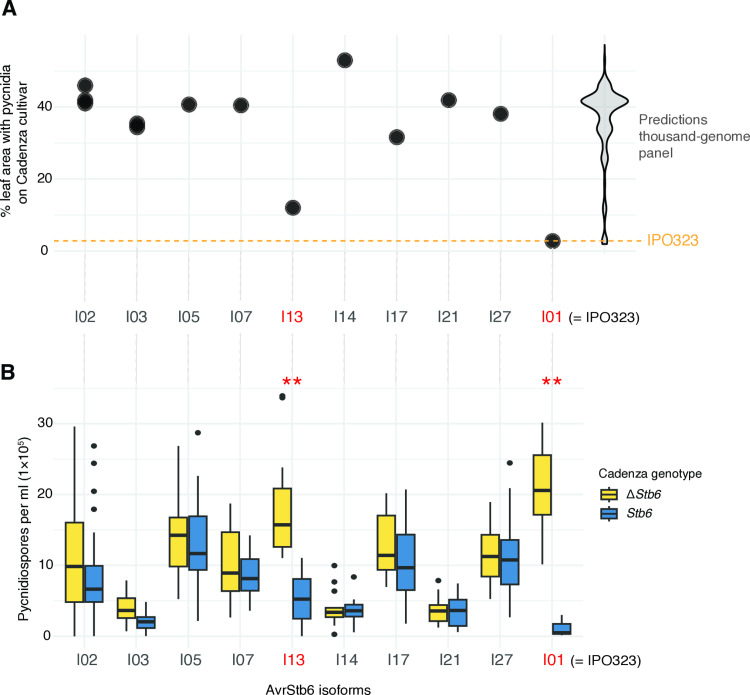
Assessment of genomic prediction accuracy with experimental data. A) Predictions based on on SNP-BLUP of a set of isolates previously analyzed by Stephens et al. (33) for virulence on isogenic lines of the Cadenza cultivar. Predictions were based on leaf area percentage covered by pycnidia on the Cadenza cultivar (carrying *Stb6*). B) Counts of pycnidiospores (spores per ml) washed off the inoculated leaves at 21 days post inoculation as determined by Stephens et al. (33). Asterisks represent isolates with highly significant (^**^ *p* < 0.005) differences in pycnidiospore counts between the resistant (*Stb6*) and the susceptible (Δ*Stb6*) isogenic lines of the Cadenza cultivar.

Even though the genomic prediction training dataset covered a substantial fraction of the global *AvrStb6* diversity, the geographic scope was limited to France. Hence, we first assessed the quality of the prediction using an independent dataset of *AvrStb6* virulence assays performed by Stephens et al. [33] and distinct from the GWAS panel. The experiments were conducted by contrasting pycnidiospore production on isogenic lines of the cultivar Cadenza carrying or not a cognate *Stb6* variant. The isolates were covering geographic regions where eight out of eleven total genetic clusters were identified in the thousand-genome panel. No isolates were originating from the Middle Eastern clusters. For the validation step, we performed single nucleotide polymorphism best linear unbiased predictions (SNP-BLUP) focused on the *AvrStb6* coding sequence to predict the percentage of leaf area containing pycnidia on the Cadenza cultivar [33] (Fig 4A). Due to the lack of full genome sequences for these isolates, genome-wide polymorphisms were not considered for the predictions. The I13 and I01 (*i.e.,* IPO323) isoforms showed the lowest virulence with <12% of leaf area containing pycnidia (Fig 4A). This contrasts with the other isoforms showing predicted percentage of leaf area containing pycnidia ranging from 26 to 53%. The prediction results align well with the experimental data gathered by Stephens et al. [33]. The isoforms I13 and I01 (*i.e.,* IPO323) were the only ones showing reduced virulence on the resistant cultivar Cadenza (*Stb6*) compared to the susceptible isogenic line (Cadenza Δ*Stb6*) (Fig 4B). Hence, the presence of *Stb6* mediates recognition in these two nearly avirulent isoforms. In contrast, all other isoforms retained virulence regardless of the Cadenza genotype consistent with these isoforms being virulent on *Stb6* (Fig 4B). These other isoforms consistently showed high percent leaf area with pycnidia on Cadenza in our predictions (Fig 4A).

After assessing the quality of the genomic predictions, we inspected best linear unbiased predictions (gBLUP) of the thousand-genome panel. We assessed the consistency of genome predictions by analyzing phenotypes predicted in isolates belonging to the GWAS panel (*i.e.,* with associated phenotypic data). Correlation coefficients for observed *vs.* predicted phenotypic values were generally worse using SNPs covering the complete genome compared to focusing the genomic prediction on *AvrStb6*-specific SNPs only (S3 and S4 Figs). This is consistent with the strong single-locus determinism of *Stb6-AvrStb6* interactions as previously reported [27]. Hence, we focused genomic predictions informed by GWAS data on green leaf area percentage on the Caphorn cultivar, identified as the trait-cultivar combination with the highest correlation coefficient, and on leaf area percentage containing pycnidia on the Cadenza cultivar, matching the cultivar used for the validation informed by the Stephens et al. [33] dataset.

**Fig 5:**
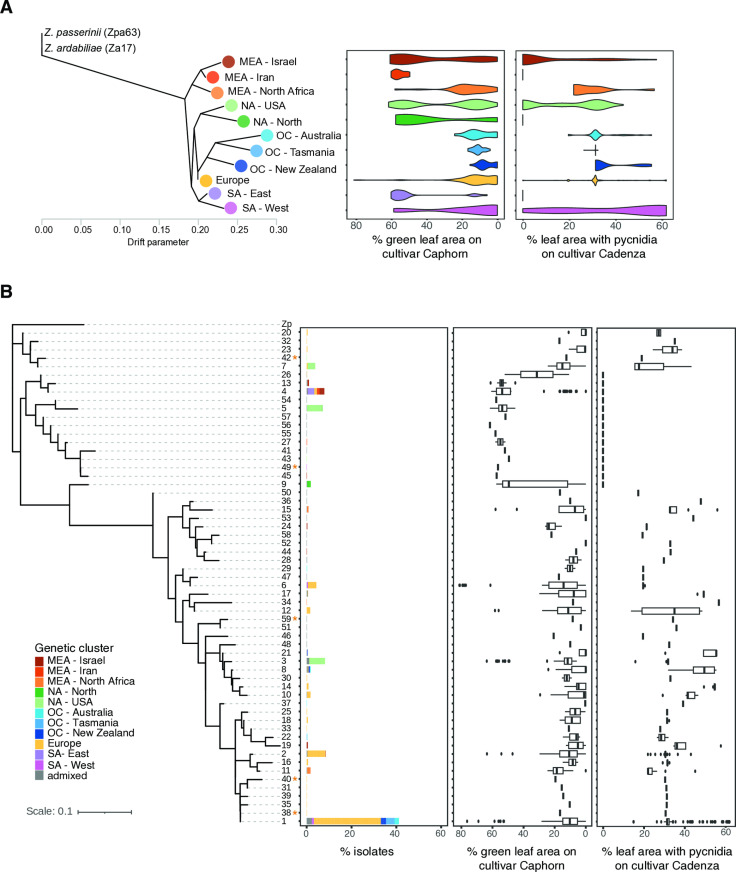
Genomic predictions for virulence on *Stb6* cultivars among the *Z. tritici* thousand-genome panel. Predictions were based on green leaf area percentage in Caphorn cultivar and leaf area percentage covered by pycnidia in Cadenza cultivar. A) Genomic predictions across population grouped by genetic cluster. B) Phylogenetic tree of AvrStb6 protein isoforms rooted based on the *Z. pseudotritici* (Zp) homolog. Truncated isoforms with premature stop codon are marked with an asterisk. Genomic predictions are summarized by AvrStb6 protein isoform.

Genotypes from the center of origin in the Middle East (Iran) were predicted to express the lowest virulence on *Stb6* cultivars, highlighted by the high green leaf area percentage on the Caphorn cultivar and the leaf area percentage with pycnidia on the Cadenza cultivar (Fig 5A). These isolates showed a higher median green leaf area percentage compared to the avirulent IPO323 isoform (isoform 6) (Fig 5B), suggesting that virulence on these cultivars can be lower than previously reported levels for avirulent isoforms. Pathogen colonization moved from the Middle East to Europe, which represented a steppingstone in the pathogen’s global dissemination to the Americas and Oceania. While most isolates from the European cluster were predicted to express high virulence on *Stb6* cultivars, a wide spectrum of virulence was observed in this cluster, with phenotypic values ranging from 0 to around 80% of green leaf area on the Caphorn cultivar and from 0 to around 60% pycnidia density on the Cadenza cultivar (Fig 5A).

Isolates from the Oceanian cluster, representing the most recently colonized continent by *Z. tritici*, were predicted to express the highest virulence on *Stb6* cultivars (Fig 5A). Conversely, isolates from the South America East cluster were predicted to be of low virulence on *Stb6* cultivars, expressing similarly low virulence on *Stb6* cultivars as isolates from the center of origin (Fig 5A). Genomic predictions across AvrStb6 isoforms showed that a trend of increasing virulence on *Stb6* cultivars with larger phylogenetic distance to the sister species homolog (Fig 5B). However, genomic predictions were showing high virulence in some of the isoforms located closest to the sister species *AvrStb6* homolog (Fig 5B). We also obtained leaf area percentage containing pycnidia predictions on the cultivar Shafir and found a high degree of correlation with the predictions for the Cadenza cultivar (S5 Fig). However, we found no predictions for fully avirulent isolates on Shafir in contrast to experimental data reporting full avirulence [27].

**Fig 6:**
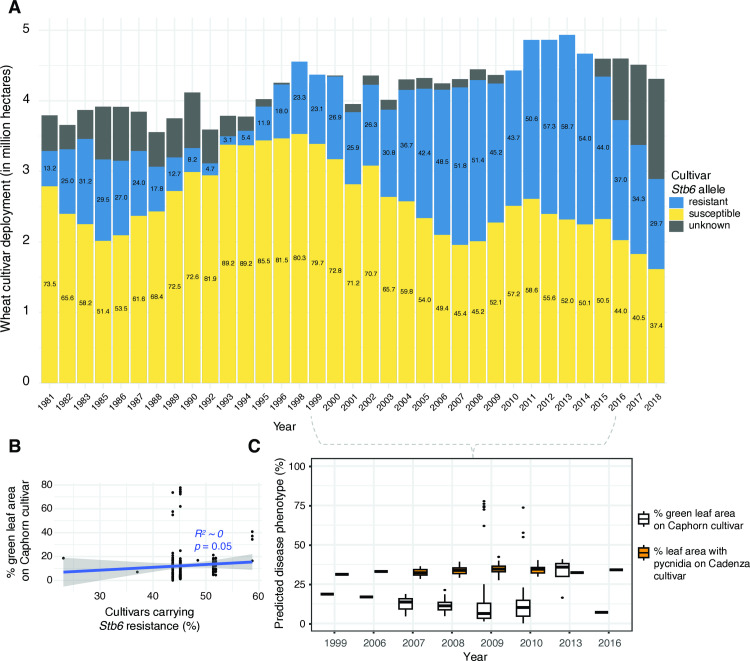
*Stb6* wheat cultivar deployment in France and genomic predictions for *Stb6* virulence. A) Registered wheat cultivar usage (in millions of hectares) as compiled by the DiverCILand database, distinguishing the percentage of cultivars carrying genotyped *Stb6* (resistant or susceptible) alleles. Numbers inside the bars indicate percentages. B) Linear regression of green leaf area genomic prediction as response of the *Stb6* resistance allele deployment in wheat cultivars across France. C) Genomic prediction of *Z. tritici* virulence on *Stb6* cultivars collected in France between 1999 and 2016 including green leaf area percentage on the Caphorn cultivar and leaf area percentage covered by pycnidia on the Cadenza cultivar.

Given the global trend of increased virulence on *Stb6* cultivars in more recently established *Z. tritici* populations, we sought to test this pattern at the regional level at high temporal resolution. France is one of Europeans largest wheat producers [38]. We genotyped 1327 French wheat cultivars for the presence of *Stb6* using diagnostic markers [39]. We then cross-referenced *Stb6* presence with yearly wheat deployment data across the country. The monitoring covered ~4–5 million hectares representing approximately 80 – 100% of total wheat cultivation in France. *Stb6* prevalence had been widely fluctuating over the 1981–2018 period of the dataset, however there was a marked increase in *Stb6* deployment starting in the mid-1990s up to the most recent years (Fig 6A). Since 2006, *Stb6* cultivars have represented almost half the wheat varieties farmed in France. We investigated whether *Z. tritici* isolates collected in France were shown trends in virulence on *Stb6* cultivars. The available samples covered the period from 1999-2016, a period with steepest increase in *Stb6* resistant allele deployment (from 23% to 52% in 2007, Fig 6A). Genomic predictions for green leaf area percentage on the Caphorn cultivar and leaf area percentage covered by pycnidia on the Cadenza cultivar showed only minor changes over years with substantial intra-year variation in predicted virulence trait expression (Fig 6C). Both predicted traits were not significantly explained by the percentage of *Stb6* resistant allele deployment in wheat cultivars across France (regression *R*^2^ ~ 0; *p*-value = 0.05; Fig 6B).

## Discussion

Plant resistance genes impose high selection pressure on fungal effector genes, causing sequence diversification or even effector deletion. *AvrStb6* is one of the best studied fungal effectors at the population level and interacts in a gene-for-gene manner with *Stb6*, a widely deployed wheat R gene [29]. By analyzing a global thousand-genome panel of the pathogen, we show that *AvrStb6* diversified strongly on the European continent with high rates of *Stb6* cultivar deployment. Diversification observation on continents with later *Z. tritici* colonization may have experienced a similar drive by *Stb6* deployment but *Stb6* remains poorly assessed outside of Europe. Consistent with this, European and Oceanian *Z. tritici* populations were also predicted to have evolved higher virulence on *Stb6* cultivars. Sequence rearrangements near effector genes can be important factors driving effector functions. We found that *AvrStb6* is flanked by a dynamic set of TEs with substantial variation across continents and multiple TEs with recent insertion success. TE activity was also the most likely cause for previously unknown *AvrStb6* losses appearing likely independently across the globe.

Rapid effector gene diversification is a convergent pathogen strategy to avoid recognition by the host immune systems [11,40]. Previous work on *AvrStb6* [27,32] and other *Z. tritici* effectors including *Avr3D1* [23] reported sequence diversity consistent with strong diversifying selection. Here, we aimed to a provide a comprehensive view on how effector diversification coincided with pathogen spread at the global scale. *Z. tritici* spread across continents in tight association with wheat cultivation [19,20]. In regions of more recent wheat cultivation and more intense production, the deployment of *Stb6*-mediated resistance has been increasing in recent decades [41]. Such increased pressure on *Z. tritici* should have caused strong diversifying selection pressure on *AvrStb6* avirulent haplotypes [27,32] including the complete replacement of the originally discovered avirulent haplotypes among most recently collected isolates [33]. Within the thousand-genome panel, we have identified 59 AvrStb6 protein isoforms, significantly expanding the previous repertoire of known isoforms [33] with the identification of 37 new isoforms, mainly considering isolates from North America. AvrStb6 isoforms in the Middle East were clustering closest to the homolog in the sister *Z. pseudotritici* homolog, consistent with the center of origin populations having retained among the most ancestral protein variants. European and Oceanian populations carried one of the most frequent but also most divergent isoforms in conjunction with the selection for virulent haplotypes of Avr*Stb6*. The same isoform has also been particularly abundant in recently collected isolates from two continents by Stephens et al. [33].

In *Z. tritici*, as in many other filamentous pathogens, effectors are located in TE-rich compartments, impacting effector and pathogenicity evolution [7,10,42–45]. Our investigation of the repetitive region surrounding *AvrStb6* revealed a complex and dynamic landscape of recent TE activity within the species. TE content underwent significant shifts as the pathogen spread from the Middle East to North-Africa and Europe, and subsequently from Europe to the Americas and Oceania. Isolates from the center of origin (Middle East – Israel and Iran) were carrying a MITE DNA transposon and a LINE retrotransposon, as the most frequent TE superfamilies upstream and downstream of *AvrStb6*, respectively. Regions outside the center of origin were predominantly carrying unclassified TEs surrounding *AvrStb6*, except for Eastern cluster in South America. TEs upstream of *AvrStb6* were consistently closer to the effector, whereas those downstream tend to exhibit greater and more variable distance to *AvrStb6* suggesting more frequent sequence rearrangements. Whether the *AvrStb6* locus has more tolerance of upstream insertion activity or whether some insertions might have been favored by selection remains unclear though. The *Z. tritici* effector *Avr3D1* also carries several TEs but of different origin and dynamic [23]. The high activity of the MITE DNA transposon was likely a consequence of the weaker apparent defenses and the tendency of MITEs to co-localize with other genes in the *Z. tritici* genome [46]. Interestingly, LINEs are completely absent in the *Z. pseudotritici* sister species genome [42], hence their rise to become the most frequent TE downstream of the *AvrStb6* locus in center of origin isolates is striking. *Z. tritici* populations tend to increase TE copy numbers throughout their global colonization history with recently established populations, such as the Americas and Oceania showing marked expansions [20,21]. However, in these recent populations, none of the most frequent TE families surrounding *AvrStb6* correspond to the TEs implicated in the strongest TE copy number expansions globally [21,22]. This shows that TE insertion dynamics near *AvrStb6* are at least partially independent from genome-wide dynamics and could be governed by selection linked to virulence on *Stb6* cultivars.

The insertion of TEs into coding sequences can result in disruption of transcription or reading frame truncation [47]. We identified five isolates (three from Europe and two from the Americas) carrying premature stop codons. However, these mutations were not caused by TE insertions directly but rather by point mutations. Previous evidence of point mutations generating stop codons in *AvrStb6* have been reported as rare events by Brunner et al. [32] in an isolate from Oregon (USA) and by Stephens et al. [33]. Beyond truncation, we identified the first evidence for low-frequency (~3%) deletions of *AvrStb6*. In two of the 34 isolates carrying no or no intact *AvrStb6*, gene loss was caused by the insertion of a TE into the coding region. The inserted TEs belonged to different superfamilies, a DNA/PIF-Harbinger transposon and a retrotransposon LINE, showing that the *AvrStb6* loss occurred at least twice independently. Interestingly, the LINE belongs to a family of TEs (ZymTri_2023_family_605) detected as one of the closest TE families downstream of *AvrStb6*, corroborating the idea that genes closer to TEs can be mutagenic [48]. *AvrStb6* loss was found distributed across different genetic clusters and continents, reinforcing the idea that the deletion of *AvrStb6* occurred multiple times. In the case of the multihost pathogen *V. dahlia*, TE insertions have also been associated with multiple independent losses of the *Ave1* effector gene, associated with an adaptive response to evade plant host immunity [16]. Despite also being located in highly plastic genomic regions, other important *Z. tritici* effector genes, *i.e., Avr3D1* and *AvrStb9*, are not known to be lost [23,49]. Earlier work suggested that *AvrStb6* may have an unknown but essential role as no losses were observed. However, our findings of multiple, likely independent *AvrStb6* deletions suggest that the gene may be dispensable for survival. Furthermore, it remains to be investigated why *AvrStb6-*mediated escape from recognition has only rarely been achieved through gene loss and instead has overwhelmingly been mediated by changes in the protein structure.

Loss of host resistance following *Stb6* cultivar deployment has been commonly observed in the wheat-*Z. tritici* pathosystem [26,31,50]. Using genomic predictions, we were able to predict virulence expression on at least some *Stb6* cultivars with reasonable accuracy as shown by using experimental data as ground truth. Even though the prediction accuracy is reasonable, the small geographic scope of the GWAS panel may well mask additional segregating genetic factors contributing to virulence in different geographies. Our findings suggest that *AvrStb6* virulence has increased as the pathogen evolved in regions where wheat cultivars carrying *Stb6* resistance gene are more widely deployed. This is particularly consistent in Europe with high rates of *Stb6* deployment. European *AvrStb6* diversity has also likely seeded diversity in haplotypes in the Americas and Oceania, allowing for further selection depending on resistance gene deployment levels. For instance, in South America (East), where *Stb6* is not widely used, isolates are predicted to have low virulence on *Stb6* cultivars. On the contrary, in Oceania, isolates are predicted to show the highest levels of virulence on *Stb6*. Using *Stb6* deployment data across France, a major wheat producing country, we found a consistent increase in deployment. The temporal resolution and sampling depth of *AvrStb6* haplotype diversity in France was not sufficient to test for clear associations with cultivar deployment. However, our worldwide data is consistent with more virulent *Z. tritici* isolates to be favored with increased *Stb6* deployment, as observed in regions such as Europe and Oceania. Overall, we show that effector locus diversification can occur rapidly and produces complex geographic and temporal patterns within a single plant pathogen species. Rapid sequence evolution of *AvrStb6* spans the spectrum of known effector modifications across different pathosystems including loss of the recognized effector, gains of virulence driven by resistance gene deployment and complex TE insertion dynamics. Our work highlights the power of large genome sequencing panels covering the known distribution range to unravel processes of rapid pathogen adaptation.

## Materials and methods

### *Z. tritici* isolates collection

We performed *AvrStb6* locus analyses on a global collection of *Z. tritici* comprising 1035 isolates analyzed by Feurtey et al. [20] (Fig 1A; S1 Table). *De novo* draft assemblies were generated by Feurtey et al. [20] using the software SPAdes v3.14.1 [51] and based on trimmed and filtered reads from Trimmomatic v0.39 [52]. To ensure acceptable *de novo* assembly qualities, we used QUAST to calculate assembly metrics [53]. We analyzed single nucleotide variants (SNVs) called by Feurtey et al. [20] to estimate genetic subdivision across the worldwide distribution range revealing 11 genetic clusters (Middle East – Israel, Middle East – Iran, Middle East – North-Africa, North America – USA, North America – North, Oceania – Australia, Oceania – Tasmania, Oceania – New-Zealand, Europe, South America – East, and South America – West), closely tracking the historic expansion of wheat cultivation around the world [20]. The genome of the closest sister species (*Z. pseudotritici*) [54] was included for phylogenetic analyses of *AvrStb6*. Additionally, we included reference-quality genome assemblies of 19 isolates representative of the global genetic diversity of the species [22] for *AvrStb6* loss-of-function analyses.

### *AvrStb6* alleles and phylogenetic analyses

We used draft genome assemblies produced by SPAdes to search for alleles of *AvrStb6* among all *Z. tritici* and *Z. pseudotritici* isolates using the *AvrStb6* IPO323 sequence (GCF_000219625.1) as query for BLASTn analyses [55]. Blastn hits were filtered for a minimum identity of 90%, minimum length (bp) match of 90% (> 330 bp), and maximum *e*-value of 10^-5^. *AvrStb6* haplotype sequences were extracted from draft assemblies based on BLASTn hit coordinates. Subsequently, the coding region sequences were aligned using MAFFT v7.520 [56], grouped into haplotypes based on 100% sequence identity and translated to amino acid sequences using EMBOSS Transeq [57]. Translated coding sequences were re-aligned using MAFFT v7.520, grouped into isoforms based on sequence identity and a maximum likelihood tree built with 1000 bootstrap replicates (MEGA11, [58]). The tree was rooted using the Z. *pseudotritici* AvrStb6 sequence. Premature stop codons leading to *AvrStb6* truncation were manually inspected in translated sequence alignments.

### Transposable elements in *AvrStb6* region

To detect TE insertions near *AvrStb6*, all Illumina scaffolds having *AvrStb6* alleles were annotated with the curated *Z. tritici* TE consensus sequences [59] using RepeatMasker v4.0.9_p2. Annotated elements shorter than 50 bp were filtered out. Since the number of TEs can vary according to the scaffold length, only the closest TEs located in an interval of 10 kb up and downstream of *AvrStb6* have been considered. TE superfamilies present in more than 50 isolates were considered as frequent. Analyzed RNA-seq expression data from a European subset of the thousand-genome panel [35] were used to assess variation in *AvrStb6* expression under minimum medium culture conditions.

### Assessing *AvrStb6* loss-of-function variants

To explore possible *AvrStb6* deletions, we first analyzed the 19-reference genome panel [22]. Specifically, for the isolate Arg00 (one of the genomes included in the panel), we analyzed raw PacBio read mapping to the *Z. tritici* IPO323 reference genome [60] using Minimap2 v2.17 [61]. Mapped reads were assembled using Canu v2.2 [62]. All assemblies were performed with an estimated genome size of 40.7 Mb (--genomeSize), error rates of 0.045 (--correctedErrorRate), minimal read length of 500 (--minReadLength) and maximum threads of 32 (--maxThreads). Alignments between the new Arg00 assemblies and IPO323 reference genome in the *AvrStb6* surrounding region were visualized using IGV v2.16.1 software [63]. Synteny was plotted with genoplotR v0.8.11 [64] for the 19-reference genome panel, using *Z. tritici* improved gene and TE annotation [36,59].

Possible *AvrStb6* deletions in the thousand-genome panel have been explored by inspecting copy number variation (CNV) calling and read count analyses of the *AvrStb6* genomic region. CNV calling was performed in the global population *de novo* draft assemblies (n = 1109) [37] from Feurtey et al. [20]. GATK CNV caller v4.1.9.0 [65] with recommended parameters on aligned BAM files has been used, with CNV interval set to 1-kb window with no overlap. Filtering criteria included GC content (min = 0.1 and max = 0.9) and removal of extremely low and high read counts [37].

### Genomic prediction for *AvrStb6*-mediated virulence

To predict *AvrStb6*-mediated virulence expressed by isolates included in the thousand-genome *Z. tritici* panel, we performed genomic prediction analysis based on the genomic best linear unbiased prediction (gBLUP) method implemented in GAPIT v3.4.0 [66]. For this, we retrieved matching phenotype-genotype datasets established for genome-wide association mapping (GWAS). We focused phenotypic data on a French collection of 103 *Z. tritici* isolates, which have been previously used to map *AvrStb6* by GWAS [27]. Phenotypes originally collected for this dataset included the percentage of green leaf area (G), percentage of necrotic leaf area (N), and percentage of leaf area containing pycnidia within the inoculated area (S). Three wheat cultivars carrying the resistance gene *Stb6* (“Cadenza”, “Shafir”, and “Caphorn”) were used for independent phenotyping assays. To evaluate the reliability of the genomic predictions, we performed a single nucleotide polymorphism best linear unbiased prediction (SNP-BLUP) analysis including a set of isolates representing nine different *AvrStb6* isoforms previously assessed for virulence on isogenic lines of the wheat cultivar Cadenza (carrying or not a cognate *Stb6*) [33]. *AvrStb6* sequences from these experimentally assessed isolates and from the thousand-genome panel were aligned using MAFFT v7.520 [58]. A maximum likelihood tree was built with 1000 bootstrap replicates (MEGA11, [58]) to cluster sequences and identify identical isoforms. Both SNP and indel variants detected in the aligned *AvrStb6* coding sequences were included for the SNP-BLUP genomic prediction analysis. To contrast genomic predictions for the percentage of leaf area containing pycnidia in the Cadenza cultivar, we used the virulence assessment previously conducted by Stephens et al. reporting pycnidiospores washed off from infected leaves [33].

As genotype input for the thousand-genome panel genomic prediction, we tested two types of SNP sets: a SNP matrix covering the *AvrStb6* region and 1000 bp up- and downstream of the effector region, and a SNP matrix covering the entire genome. The SNP matrices were generated by filtering for biallelic SNPs (option “M2”) and a minor allele frequency of 5% (-q 0.05: minor) using BCFtools v1.5 [67]. The kinship matrix was calculated using plink v1.90 [68] based on genome-wide SNPs. We used vcftools [69] “--thin 1000” to randomly retain only 1 SNP for every 1 kb interval as an input dataset for the calculation of the kinship matrix.

Given that input leaf area phenotypic trait values were measured in percentage (ranging from 0-100%), any phenotypic prediction values falling below 0 and above 100 were adjusted to 0 and 100, respectively. Pearson correlation coefficients between phenotypic traits measured in the French collection versus predicted phenotypic values in the same population were calculated in R v4.3.2 using the *cor* function. Correlation coefficients were used to select the best combination of cultivars and traits from the 9 possible pairings, as well as SNP matrix for performing genomic predictions.

### *Stb6* wheat deployment in France and *Stb6* genotyping

Wheat cultivar deployment data for France were retrieved from the DiverCILand database (https://wheat-diverciland.moulon.inrae.fr/). DiverCILand aims to monitor varietal diversity at various scales and gathers data on the deployment areas of registered bread wheat varieties for 17 European countries. Depending on the country, the data covers 5–30 years of deployment history either at the county or national scale. The data on cultivar deployment in France were produced by FranceAgriMer and covers the period 1981–2018.

*Stb6* genotyping of French cultivars was performed using KASP genotyping assays following manufacturer instructions (LGC Genomics) and diagnostic markers cfn80047 and cfn80050 [39]. The run cycle and data analysis were performed on the LightCycler 480 Real-Time PCR System (Roche Life Science). Knowledge on *Stb6* allelic variants (*i.e.,* resistant or susceptible) were also retrieved from the genotyping data of 4506 wheat accessions [70] using diagnostic marker AX-89415184 [71]. The percentage of cultivars carrying *Stb6* resistance or susceptible alleles per year was calculated on 1327 French cultivars having both cultivar deployment and *Stb6* allelic variant information.

Predicted phenotypic values of 211 French *Z. tritici* isolates, collected between 1999 and 2016 were inspected in detail. To determine whether the phenotypic traits observed in the French collection could be significantly explained by the frequency of Stb6-resistant allele deployment in wheat cultivars across France, a linear regression analysis was performed using the *lm* function in R v4.3.2.

## Supporting information

S1 Fig Genetic clusters relative frequency per protein isoform. (TIF)

S2 Fig Visualized mapped PacBio long reads produced from isolate Arg00 mapped to the IPO323 reference genome centered on AvrStb6. AvrStb6 is located at 69019–69383 bp.
(TIF)

S3 Fig Correlation plots between phenotypic data collected on the mapping population and predicted phenotypic values based on a SNP matrix covering the AvrStb6 region and 1000 bp up and downstream of the effector. Correlations were assessed among all three traits (G - green leaf area percentage, N - necrotic leaf area percentage, S - leaf are percentage containing pycnidiospores within the inoculated area) across the three wheat cultivars (CAD – Cadenza; SHA – Shafir; CAP – Caphorn). *R* values indicate the Pearson correlation coefficient between phenotypic data and predicted values for the same isolates.
(TIF)

S4 Fig Correlation plots between phenotypic data collected on the mapping population and predicted phenotypic values based on a SNP matrix covering the entire genome. Correlations were assessed among all three traits (G - green leaf area percentage, N - necrotic leaf area percentage, S - leaf are percentage containing pycnidiospores within the inoculated area) across the three wheat cultivars (CAD – Cadenza; SHA – Shafir; CAP – Caphorn). *R* values indicate the Pearson correlation coefficient between phenotypic data and predicted values for the same isolates.
(TIF)

S5 Fig Genomic predictions of leaf area percentage covered by pycnidia on the Shafir cultivar. Phylogenetic tree of AvrStb6 protein isoforms rooted based on the Z. pseudotritici (Zp) homolog. Truncated isoforms with premature stop codon are marked with an asterisk. Genomic predictions are summarized by AvrStb6 protein isoform.
(TIF)

S1 Table Analyzed Zymoseptoria tritici collection (n=1035), including isolate name, geographical location of sampling site, assigned population cluster, and sampling date. Retrieved from Feurtey et al. [20].
(XLSX)

S2 Table Zymoseptoria tritici draft genome assemblies with detected AvrStb6 homologues, respective scaffold location of the homologue, BLAST hit statistics, haplotype and isoform number, assembly N50. (XLSX)

S3 Table *AvrStb6* isoform identifiers and protein sequences. Where matching, isoform identifiers from Stephens et al. [33] were added.
(XLSX)

S4 Table Protein isoforms with premature stop codon with the respective amino acid position. (XLSX)

S5 Table Most frequent transposable elements (TEs) detected near *AvrStb6*, their respective family number, superfamily name, and distance to *AvrStb6.* (XLSX)

S6 Table Isolates lacking AvrStb6 homologues, BLAST hit statistics, presence of copy-number variation, mapped read count in AvrStb6 ORF, N50 draft assembly. (XLSX)
