# Supplementary figures and images for: Diversification, loss, and virulence gains of the major effector AvrStb6 during continental spread of the wheat pathogen Zymoseptoria tritici
Source: PLoS Pathog. 2025 Mar 31;21(3):e1012983. doi: 10.1371/journal.ppat.1012983 (PMC11984979; doi:10.1371/journal.ppat.1012983)

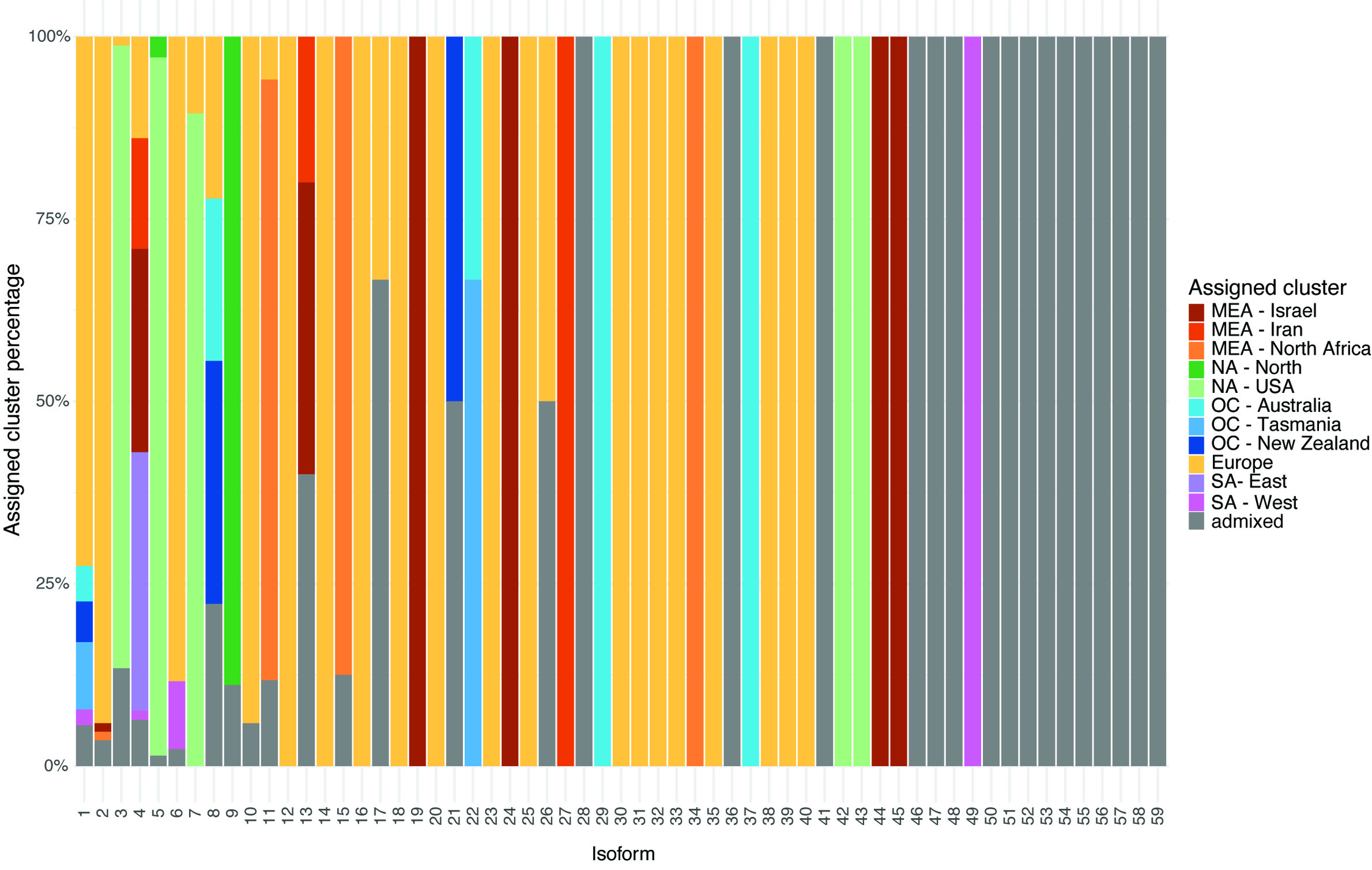

Supplement: S1 Fig — (TIF) [file ppat.1012983.s001.tif]

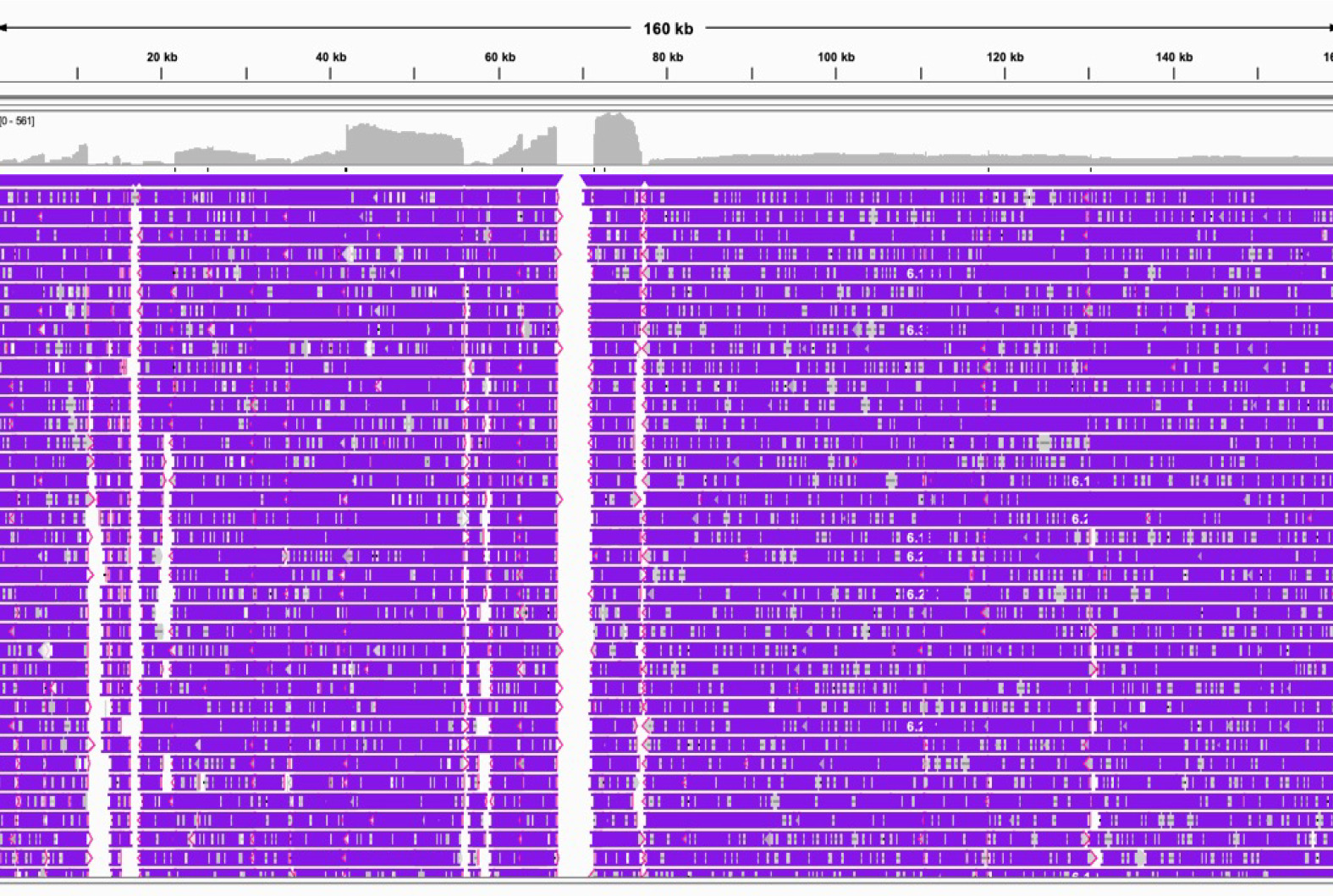

Supplement: S2 Fig — AvrStb6 is located at 69019–69383 bp. (TIF) [file ppat.1012983.s002.tif]

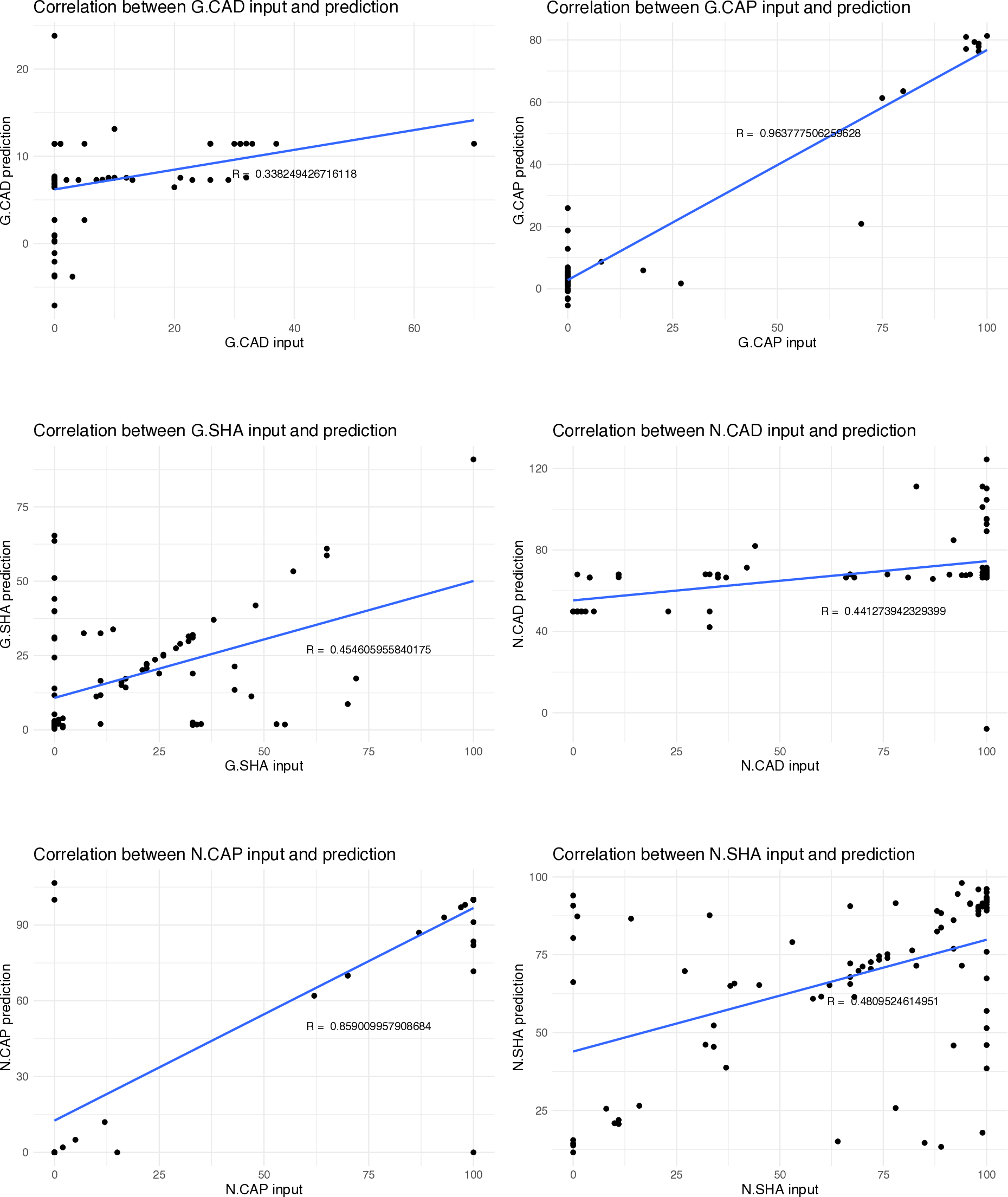

Supplement: S3 Fig — Correlations were assessed among all three traits (G - green leaf area percentage, N - necrotic leaf area percentage, S - leaf are percentage containing pycnidiospores within the inoculated area) across the three wheat cultivars (CAD – Cadenza; SHA – Shafir; CAP – Caphorn). R values indicate the Pearson correlation coefficient between phenotypic data and predicted values for the same isolates. (TIF) [file ppat.1012983.s003.tif]

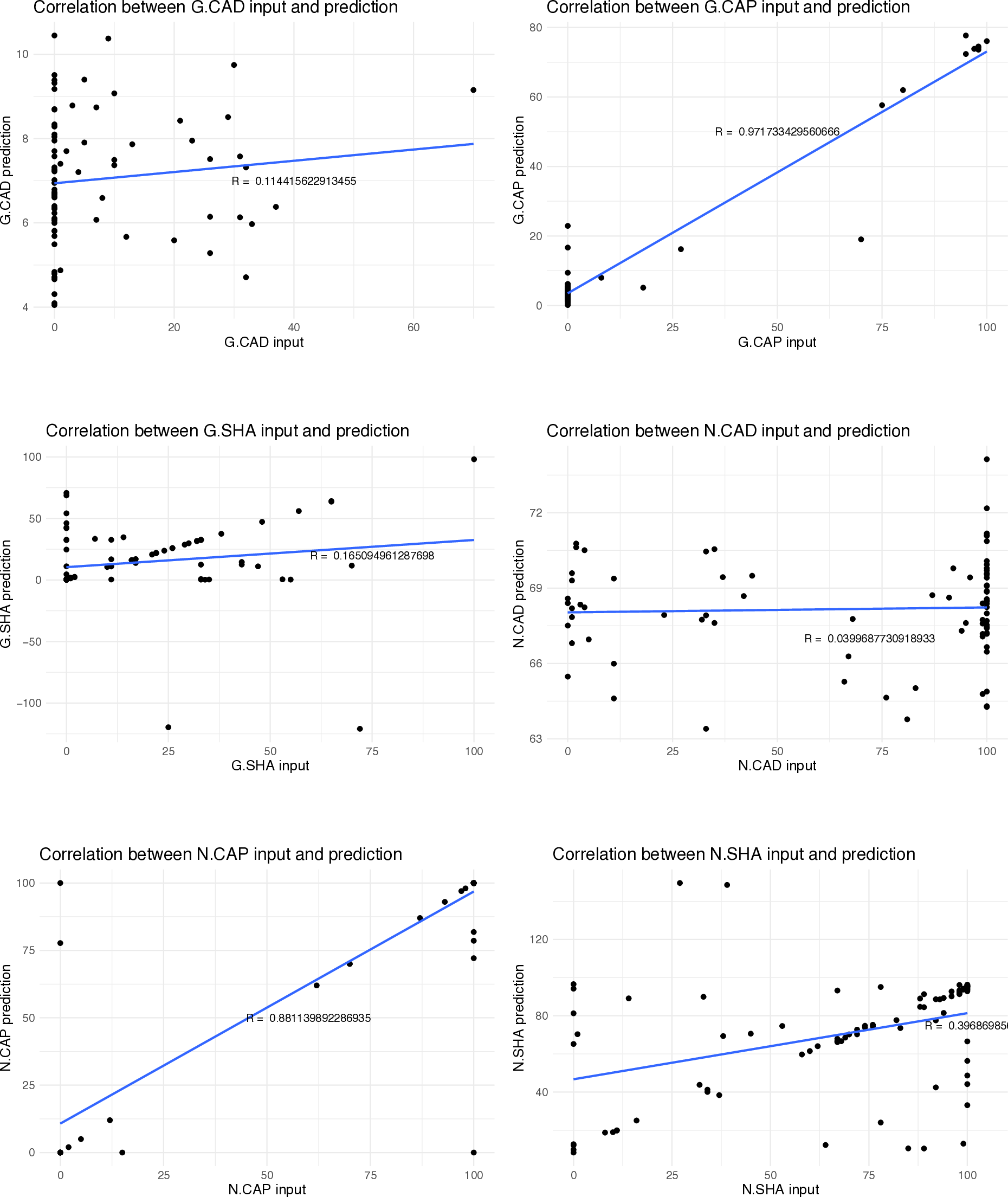

Supplement: S4 Fig — Correlations were assessed among all three traits (G - green leaf area percentage, N - necrotic leaf area percentage, S - leaf are percentage containing pycnidiospores within the inoculated area) across the three wheat cultivars (CAD – Cadenza; SHA – Shafir; CAP – Caphorn). R values indicate the Pearson correlation coefficient between phenotypic data and predicted values for the same isolates. (TIF) [file ppat.1012983.s004.tif]

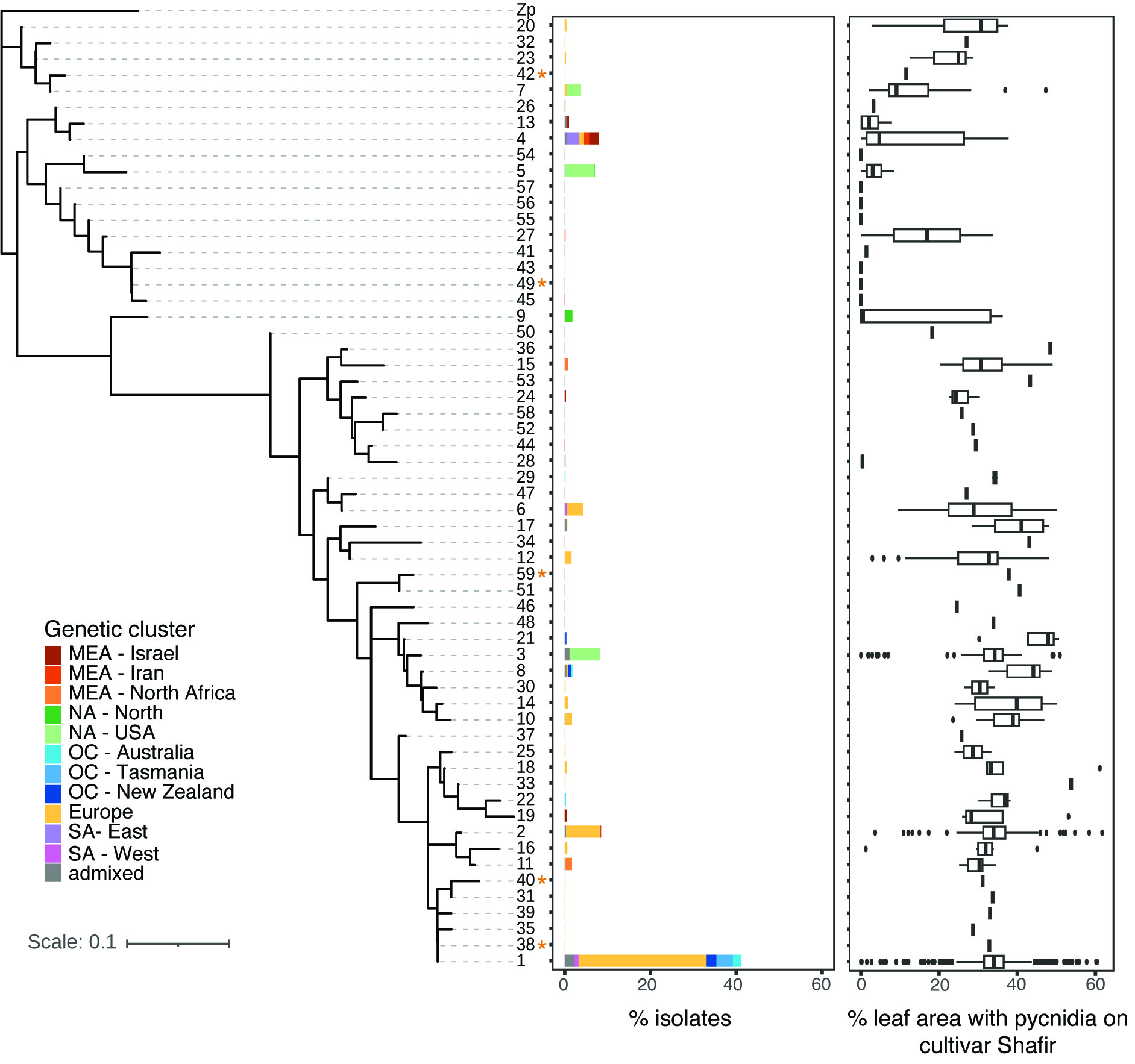

Supplement: S5 Fig — Phylogenetic tree of AvrStb6 protein isoforms rooted based on the Z. pseudotritici (Zp) homolog. Truncated isoforms with premature stop codon are marked with an asterisk. Genomic predictions are summarized by AvrStb6 protein isoform. (TIF) [file ppat.1012983.s005.tif]
